# The effect of various weight-bearing activities on the motion of lumbar facet joints in vivo

**DOI:** 10.1186/s13018-022-03016-5

**Published:** 2022-02-21

**Authors:** Wangqiang Wen, Haoxiang Xu, Zepei Zhang, Bowen Kou, Quan Sun, Jun Miao

**Affiliations:** 1 Graduate School, Tianjin Medical University, Tianjin, China; 2 Department of Spine Surgery, Tianjin Hospital, Jiefangnanlu 406, Hexi District, Tianjin, 300211 China; 3 The Second Affiliated Hospital of Guangzhou University of Chinese Medicine, Guangzhou, Guangdong China

**Keywords:** Lumbar facet joints, Kinematics, Range of motion, Loads, Symmetry, Body position

## Abstract

**Background:**

Lumbar facet joints (LFJs) are usually related to the pathogenesis of the spine. The purpose of this paper is to study the effects of lifting load on the motion of lower lumbar facet joints in vivo.

**Methods:**

Ten healthy volunteers aged 25 ≤ 39 years, 5 males and 5 females, were recruited. Using a dual fluoroscopy imaging system (DFIS) combined with CT, firstly, the L3-S1 segment image scanned by CT was converted into a three-dimensional model. Then, the lumbar motion images of L3-S1 vertebrae taken by the DFIS under different loads (0 kg, 5 kg, 10 kg) and different body postures (maximum flexion and extension, maximum left and right bending, and maximum left and right torsion) were captured. Finally, in the Rhino software, the instantaneous motion state of the lumbar spine is reproduced by translation and rotation according to the anatomical structure of the lumbar spine and the previous images. With the help of computer software, a Cartesian coordinate system was placed in the center of each articular surface to measure the kinematics of the articular process and to obtain 6DOF data under different loads (0 kg, 5 kg, 10 kg) in the lumbar facet joints.

**Results:**

In the flexion and extension of the trunk, weight bearing reduced the translational range in the mid-lateral direction. In the L3/4 segment, the lateral translational range of the left and right facet joints gradually decreased with increasing load, and the translational range at 0 kg was significantly greater than that at 10 kg (left side: 0 kg, 0.86° ± 0.57°, 10 kg, 0.24° ± 0.26°, *p* = 0.01; right side: 0 kg, 0.86° ± 0.59°, 10 kg, 0.26° ± 0.27°, *p* = 0.01). In the L5/S1 segment, the translation range of the LFJ at 0 kg was significantly greater than that at 10 kg (*p* = 0.02). Other bending and rotation movements were not found to cause differential changes in the 6DOF of the LFJ. In bending, the rotation range was the largest in the L3/4 segment (*p* < 0.05) and gradually decreased from top to bottom. At the same level, there were significant differences in the translation range of the left and right facets in the anterior posterior and craniocaudal directions (*p* < 0.05).

**Conclusion:**

Increasing the load has a significant impact on the coupled translational movement of lumbar facet joints. The asymmetric translational movement of the left and right facet joints may be a factor that accelerates the degeneration of facet joints.

## Introduction

Facet joints play an important role in the stability and movement of the entire spine [1]. In addition to restricting the movement of the vertebrae, the main function of joints can also bear different types of loads, such as compression, stretching, shearing, and torsion [2, 3]. Lumbar facet joint osteoarthritis is also considered the main cause of chronic low back pain in 15–45% of patients [4–6]. Heavy lifting (over 25 kg) and trunk flexion and rotation are considered to be moderate risk factors for low back pain [7, 8].

From a clinical point of view, an accurate description of the kinematics of normal lumbar facet joints can help us to improve our understanding, diagnosis and treatment of degenerative spine diseases. A literature review shows that data reports on the motion patterns of lumbar facet joints are mainly based on cadaver models [3], animal models [9, 10], computed tomography (CT) [11–13] and magnetic resonance imaging (MRI) [14] had been gradually applied to describe the morphology of the lumbar spine and the classification of osteoarthritis. The dual fluoroscopy imaging system DFIS is one of the latest technologies used to study the range and direction of motion of small joints in vivo.

This technology had been widely used in various bone, spine and joint studies. There are no restrictions on the subjects' activities, which overcomes the limitations of MRI/CT in space, time and body position, and can obtain the in vivo kinematics data of different weight-bearing and activity states under the real physiological conditions of the human body. In terms of LFJs, Li and his colleagues [15] were the first to use this technique to quantify the in vivo kinematics of lumbar facets in nonfunctional weight-bearing positions. Yin [16] also studied the effect of degenerative facets on the range of motion of the lumbar spine through this system. Although these studies have greatly analyzed the kinematics of LFJs in vivo under functional loading, their research direction is limited to either non-weight bearing or flexion and extension. Because the movement of the spine is complex, most daily life and work require complex movements such as flexion, lateral bending and rotation under appropriate weight bearing. Thus, it is very important to understand the in vivo kinematics of LFJs under different postures and various loads. These data are of great significance to study the mechanism of delaying the disease progression of the lower lumbar spine.

Therefore, the purpose of this paper is to study the effects of lifting load (0 kg, 5 kg, 10 kg) on the flexion and extension, left–right bending and left–right rotation of lower lumbar facet joints. We used the 3D modeling method of a dual fluoroscopy imaging system (DFIS) combined with CT to measure the facet motion of the lumbar L3-S1 vertebral body. This technique has been validated by our team for accuracy and repeatability and can be used to noninvasively measure spinal motion during weight-bearing functional activities [17]. We hypothesize that the in vivo kinematics of lumbar facets are affected by load materials and methods.

## Methods

### Participants

In this study, 10 healthy asymptomatic volunteers, 5 males and 5 females aged between 25 and 39 years, were recruited. The inclusion criteria were (1) normal spine development and lumbar mobility within the normal range and (2) no previous symptoms of low back pain and history of lumbar trauma and sprain. (3) BMI was within the normal range, 18.5 < BMI < 25, and (4) bone mineral density was normal. The exclusion criteria were (1) previous symptoms of low back pain, history of low back trauma and sprain, (2) spinal deformities, (3) currently pregnant, (4) BMI > 25 or BMI < 18.5, and (5) Pfirrmann disk degeneration classification > grade II. This study was approved by the medical ethics committee of Tianjin Hospital.

### Original CT image acquisition of lumbar spine

**Fig. 1:**
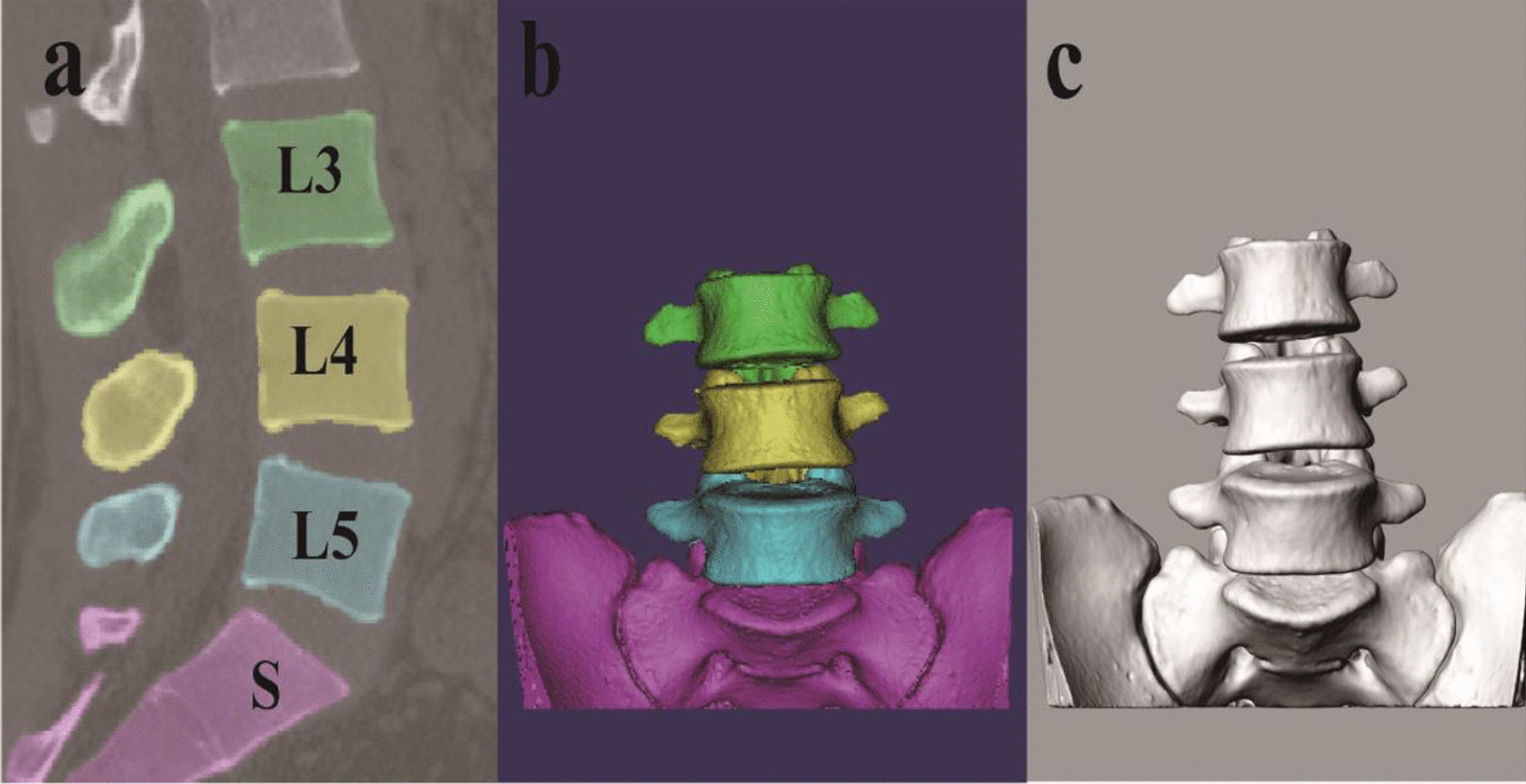
**a** Import the DICOM file into MIMICS, and select the L3-S1 cone. **b** Build L3-S1 vertebra in mimics. **c** Establish L3-S1 vertebral body in rhino software

All subjects were scanned in a supine and relaxed position using computed tomography (CT) (Sensation 16 Siemens, Germany). The subjects were instructed to avoid any aggressive activities within 2 h before the experiment and rest on the examination table for approximately 20 min before CT scanning. The high-resolution axial plane image was scanned to obtain a parallel digital image with a thickness of 0.625 mm and a resolution of 512 * 512 pixels, and the CT image was saved in medical digital imaging and communication format (DICOM). The DICOM images were imported into solid modeling software (mimics version 19.0), and the 3D anatomical vertebral body model of L3-S1 was constructed using the established and validated protocol (Fig. 1a, b) [17]. After outputting the 3D model in binary stereolithography (STL) format, it was imported into solid modeling software (rhinoceros® Robert McNeel & Associates, Seattle, Washington). The vertebral bodies were organized into a group to establish a 3D model of the L3-S1 vertebral body (Fig. 1c).

### Dual fluoroscopy imaging system

**Fig. 2:**
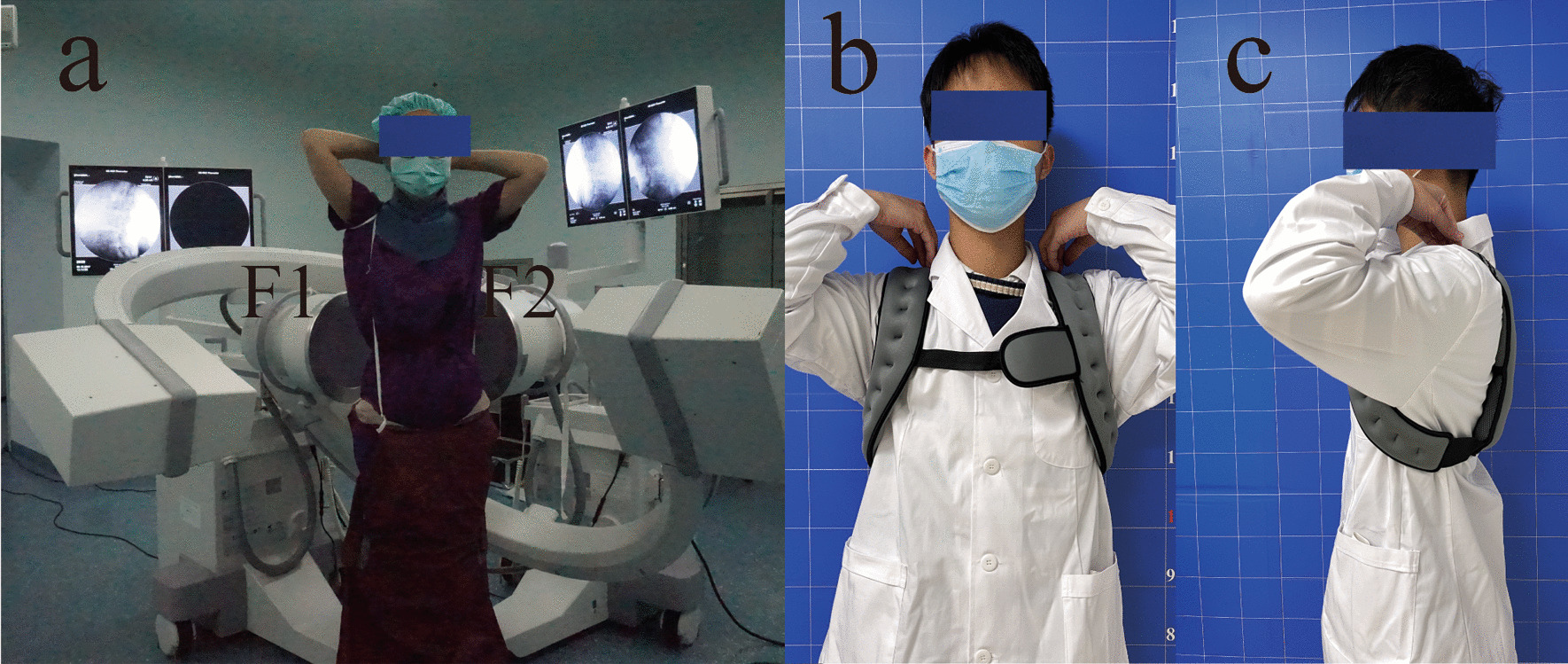
**a** Dual fluoroscopy imaging system, **F1** and **F2** are mutually perpendicular disks. **b**, **c** Spinal weight-bearing method with unlimited movement

The dual fluoroscopy imaging system consists of two "C" arm x-ray machines of the same model. Two fixed "C" arm x-ray machines were placed in the vertical position (Fig. 2a. F1, F2) to capture lumbar images in different postures from two orthogonal directions at the same time. Under the guidance of two spinal surgeons, the subject's lumbar spine was observed to ensure that it is always within the cross-projection range of the two "C" arms during movement and that the lumbar L3-S1 segment is basically within the field of vision of two fluoroscopes. Each subject was required to perform maximum flexion to maximum extension, maximum left to right bending, and maximum left to right rotation at loads of 0 kg, 5 kg (carrying 5 kg sandbags) and 10 kg (carrying 5 kg sandbags at the front and rear). The sandbag is located on the chest and back to avoid restricting waist movement (Fig. 2b, c). Each image acquisition position was maintained for approximately 2 s to obtain an instantaneous x-ray double oblique perspective image. During this period, the researchers assisted in restraining the hip and knee joints to reduce errors caused by other joint activities, and the subjects wore custom lead clothes to protect their thyroids and gonads (Fig. 2a).

### Three-dimensional reconstruction of lumbar facet joint motion

**Fig. 3:**
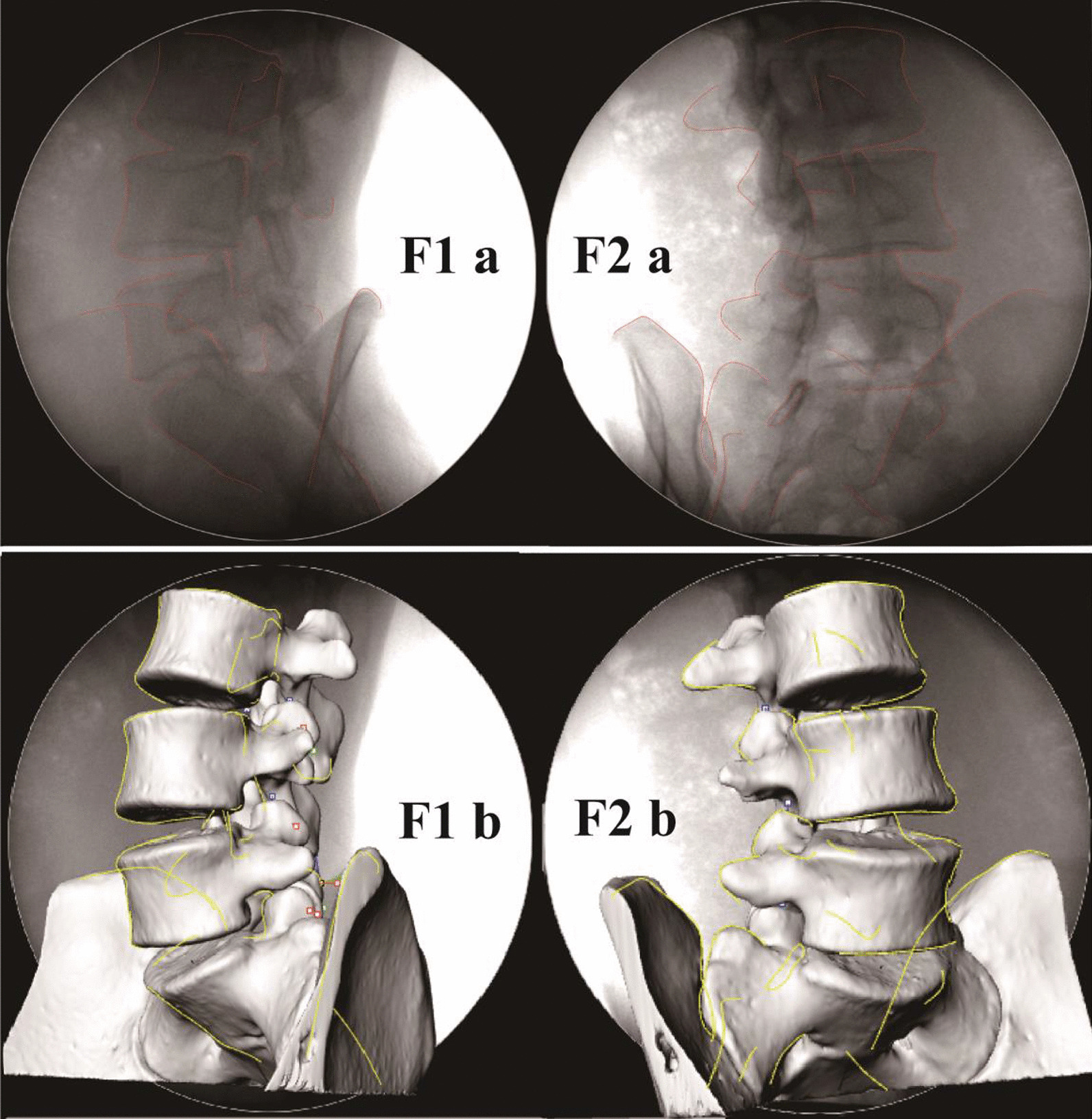
**F1a, F2a**, outline the vertebral structure in rhino software. **F1b and F2b**, through the translation and rotation of the vertebral body, make the vertebral body fully match the perspective image, and realize two-dimensional and three-dimensional matching

The in vivo position of the vertebral body in different weight-bearing positions can be reproduced in the modeling software with DFIS [17]. The three-dimensional model obtained in previously was imported into the modeling software (Rhino), then we outlined the anatomical characteristics of the lumbar spine in each posture, which must to be outlined the significant structures such as vertebral body, superior and inferior articular process and spinous process in detail (Fig. 3, F1a, F2a). In Rhino, through vertebral fluoroscopy the translation and rotation of the three-dimensional vertebral model, the spatial position of the lumbar spine was adjusted so that the three-dimensional lumbar model could match two mutually perpendicular double-oblique x-ray fluoroscopy images at the same time to realize the matching of two-dimensional and three-dimensional images (Fig. 3, F1b, F2b). The above can ensure the accuracy of matching results to a great extent to reduce the penetration of the model. Another important way to reduce model penetration and ensure data scientificity is that this series of test processes require system calibration. According to the C-arm used in the research, we had specially made the calibration system instrument for DFIS and compiled the corresponding correction processing software program [17]. The repeatability of this technique in reproducing the 6DOF kinematics of the human spine in vivo is less than 0.3 mm in translation and less than 0.7 degrees in rotation [17, 18]. This technique can reproduce the position of the vertebral body in different postures. By matching the L3-S1 standard model, we reproduced the in vivo motion of lumbar facet joints under different loads (0 kg, 5 kg, 10 kg) and different postures.

### Calculation of motion range of lumbar facet joint

**Fig. 4:**
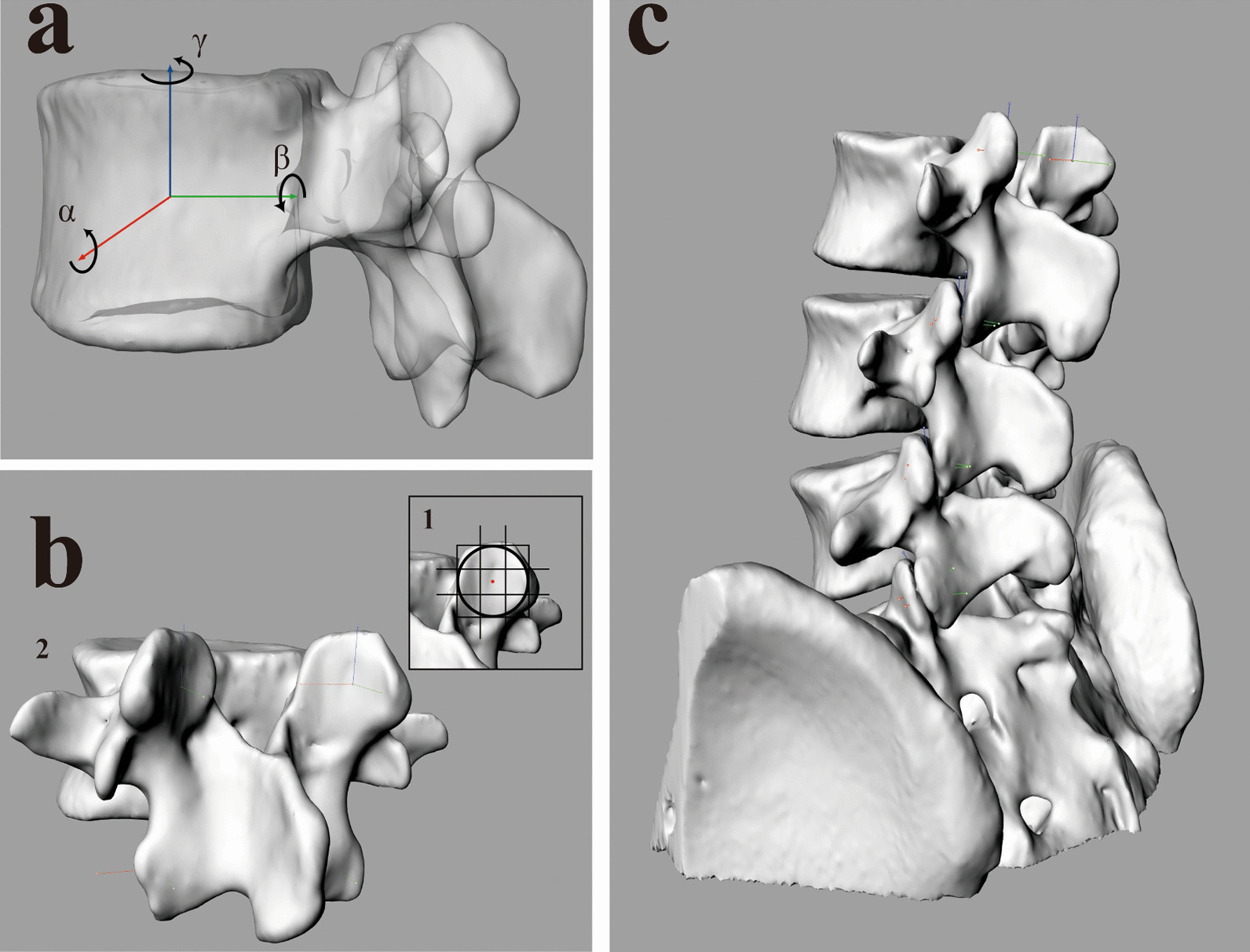
**a** Establish a Cartesian coordinate system at the center of the vertebral body. **b**, **c** place the Cartesian coordinate system established above in the center of the joint capsule. α The range of rotation around the mid-lateral axes, β Rotation range around the anteroposterior axes, γ The range of rotation around the craniocaudal axis

In the L3-S1 model obtained in the supine position, a right-hand Cartesian coordinate system was established in the center of the vertebral body (Fig. 4 a). The articular surface of the articular process of the target vertebral body were divided into nine parts. The center was designated as the volume center of the facet capsule (Fig. 4 b1). The facet capsule coordinate system can be established by moving the coordinate system to the center point (Fig. 4 b2), which could calculate the relative activity data of two adjacent joints (Fig. 4c). The X axis (red) was defined as perpendicular to the sagittal plane of the cone and pointing to the left, the Y axis (green) was defined as parallel to the sagittal plane of the cone and pointing to the back, and the Z axis (blue) was defined as perpendicular to the coronal plane of the cone and pointing to the left cephalic side.

The ROM size of LFJ was divided into rotation angle and space vector size. The rotation angles along the X, Y and Z axes were α, β and γ, which represents the rotation range of the vertebral body in the movement process. The X (red), y (green) and Z (blue) axes in the spatial coordinates represent the direction and the vector size in space, respectively (Fig. 4a). The angle and translation of lumbar facet joint movement were calculated by the range of motion difference method. The final difference is taken as the absolute value to observe the range of motion in the facet joint.

### Statistical analysis

Two-way repeated-measures ANOVA was used to compare the range of motion of the lumbar facet joints at the L3/4, L4/5 and L5/S1 vertebral levels under weight-bearing conditions. Kinematics was the dependent variable, and weight bearing and vertebral body level were the independent variables. When a statistically significant difference was detected, a post hoc Newman–Keuls test was performed, and the level of significance was again chosen at *p* < 0.05. A paired t test was used to compare the difference in bilateral facet joint displacement. If *p* < 0.05, it was statistically significant. All analyses were performed using SPSS software (SPSS 19.0; IBM, Armonk, NY, USA), with continuous variables expressed as X ± S.

## Results

**Fig. 5:**
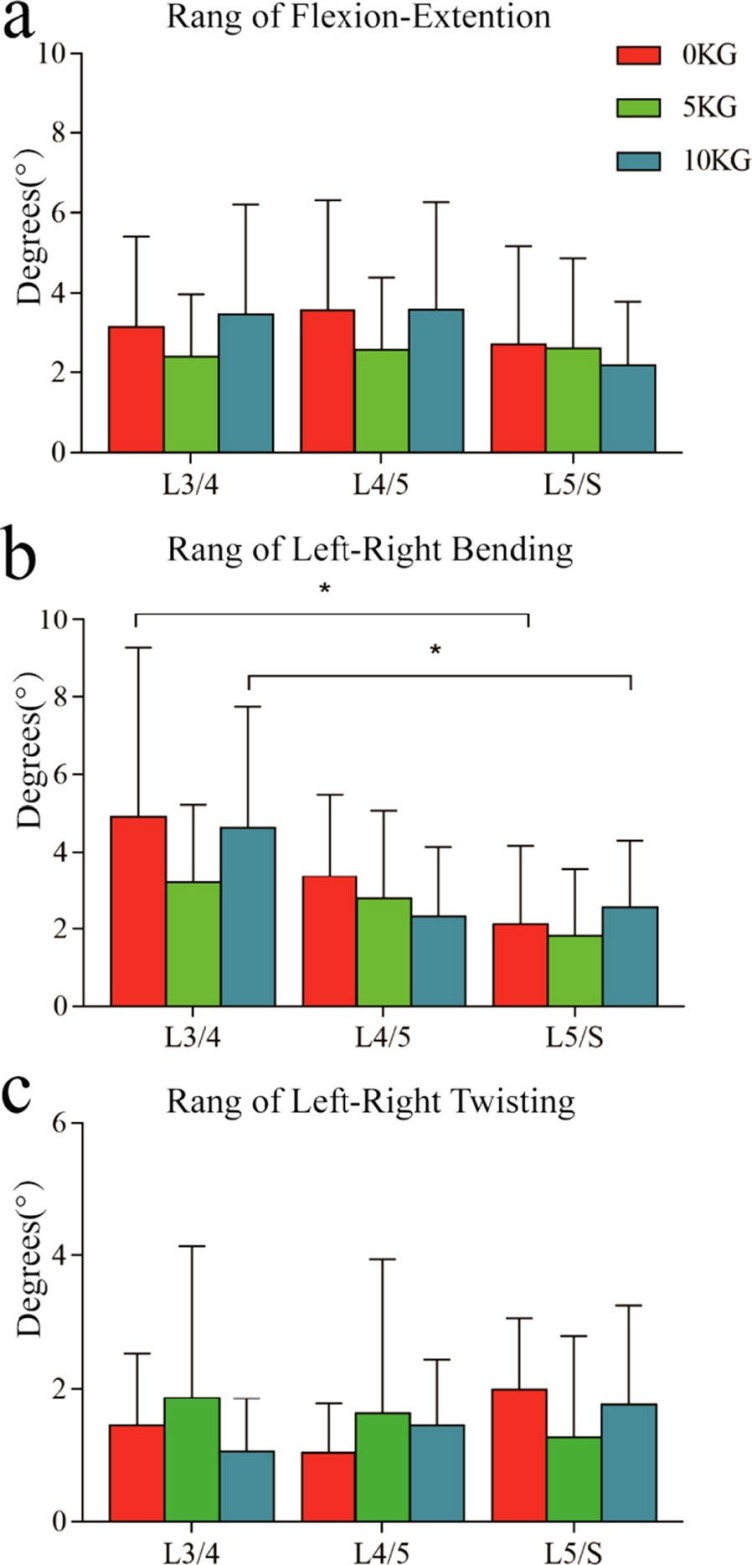
Primary rotation range of facet joint. **a** Maximum flexion to maximum extension. **b** maximum left–right bending. **c** maximum left–right torsion of trunk. The symbol (*) represents the statistical significance of the comparison between levels (*p* < 0.05)

In the flexion and extension of the trunk, the lumbar facet joint mainly rotated around the medial and lateral axes. The average rotation ranges of L3/4, L4/5 and L5/S1 levels under load were 2.72° ± 2.45° to 3.58° ± 2.73°, 2.58° ± 1.81° to 3.59° ± 2.68°, and 2.16° ± 1.63° to 2.72° ± 2.45°, respectively. The load condition had no significant effect on the rotation range of the main rotating shaft and the coupling shaft (*p* > 0.05), and the data showed that there was no significant difference in the rotation range of the main rotating shaft in different horizontal segments (Fig. 5a). The translation direction was mainly craniocaudal, ranging from 1–2.5 mm. The load had no effect on it. Interestingly, for the coupled translation, we found that the load reduced the translation in the mid-lateral direction. In the L3/4 segment, the translation range of the left and right facets under 0 kg load was greater than under 5 kg and 10 kg and significantly greater than that under a 10 kg load (left: 0 kg, 0.86 ± 0.57°, 10 kg, 0.24 ± 0.26°, *p* = 0.01; right: 0.86° ± 0.59°, 0.26° ± 0.27°, *p* = 0.01). In the L5/S1 segment, the 0 kg load translation range of the left facet was significantly greater than the 10 kg load translation range (*p* = 0.02). In addition, when the left and right facets at the same level were loaded with 0 kg, the translation range of the L4/5 segment in the anteroposterior direction was greater on the left than on the right (*p* < 0.05). When loading 10 kg, the translation range of the L3/4 segment in the craniocaudal direction was greater on the left than on the right (*p* < 0.05) (Table 1).

**Table 1:** Comparison of the difference between left and right joint translation coupling process

Position	Level	Loads	Left side joint translation (mm)			Right side joint translation (mm)		
			**X**	**Y**	**Z**	**X**	**Y**	**Z**
FL-EX	L3/4	0 KG	0.86 ± 0.57	0.44 ± 0.49	1.8 ± 1.58	0.86 ± 0.59	0.86 ± 0.45	1.7 ± 1.16
		5 KG	0.61 ± 0.33	0.49 ± 0.37	1.38 ± 1.02	0.6 ± 0.34	0.69 ± 0.51	1.07 ± 0.84
		10 KG	0.24 ± 0.26	0.55 ± 0.38	1.92 ± 1.41*	0.26 ± 0.27	0.9 ± 0.78	1.19 ± 1.08*
	L4/5	0 KG	0.71 ± 0.69	1.55 ± 0.83*	1.6 ± 1.14	0.71 ± 0.68	1.05 ± 0.42*	1.76 ± 1.27
		5 KG	0.5 ± 0.34	0.64 ± 0.61	1.18 ± 1.03	0.49 ± 0.35	0.71 ± 0.73	1.26 ± 0.76
		10 KG	0.62 ± 0.51	0.73 ± 0.45	1.42 ± 1.05	0.68 ± 0.5	1.01 ± 1.09	1.41 ± 1.14
	L5/S	0 KG	0.85 ± 0.45	0.99 ± 0.95	2.05 ± 1.71	0.83 ± 0.44	0.99 ± 1.19	2.1 ± 1.75
		5 KG	0.7 ± 0.5	0.85 ± 0.46	1.29 ± 1.07	0.71 ± 0.46	1.29 ± 0.78	1.54 ± 1.35
		10 KG	0.47 ± 0.16	1.35 ± 0.72	1.79 ± 1.11	0.51 ± 0.18	1.36 ± 0.9	1.76 ± 1.54
LB-RB	L3/4	0 KG	0.75 ± 0.58	0.55 ± 0.32	1.81 ± 1.42	0.8 ± 0.57	0.64 ± 0.32	1.43 ± 1.15
		5 KG	0.88 ± 1.03	0.99 ± 0.6*	1.64 ± 1.02*	0.69 ± 0.42	0.42 ± 0.33*	0.55 ± 0.65*
		10 KG	0.41 ± 0.46	0.91 ± 0.58	1.02 ± 0.53	0.53 ± 0.45	0.76 ± 0.57	0.76 ± 0.78
	L4/5	0 KG	0.68 ± 0.48	0.98 ± 0.6	0.83 ± 0.63	0.59 ± 0.43	0.64 ± 0.36	1.19 ± 0.78
		5 KG	0.6 ± 0.46	0.5 ± 0.38	0.99 ± 0.77	0.56 ± 0.37	0.58 ± 0.43	1.23 ± 1
		10 KG	0.69 ± 0.63	0.6 ± 0.48	1.38 ± 0.82	0.73 ± 0.67	0.55 ± 0.34	0.88 ± 0.49
	L5/S	0 KG	0.64 ± 0.25	0.9 ± 0.51	1.24 ± 1.14	0.68 ± 0.23	1.09 ± 0.69	1.18 ± 1.12
		5 KG	0.82 ± 0.79	0.89 ± 0.94	0.99 ± 0.75	0.75 ± 0.72	1.04 ± 0.65	1.13 ± 0.75
		10 KG	1.27 ± 1.36	0.82 ± 0.56*	1.3 ± 0.99	1.3 ± 1.44	1.89 ± 1.34*	1.47 ± 0.75
LT-RT	L3/4	0 KG	0.63 ± 0.44	0.41 ± 0.27	1.07 ± 0.75	0.68 ± 0.48	0.47 ± 0.41	0.68 ± 0.41
		5 KG	0.51 ± 0.56	0.48 ± 0.3*	1.05 ± 0.72	0.52 ± 0.52	0.81 ± 0.45*	1.12 ± 0.87
		10 KG	0.61 ± 0.58	0.66 ± 0.56	0.95 ± 0.54	0.63 ± 0.57	0.62 ± 0.33	1.31 ± 0.97
	L4/5	0 KG	0.57 ± 0.39	0.44 ± 0.44	1.12 ± 1.12	0.59 ± 0.41	0.57 ± 0.26	0.73 ± 0.62
		5 KG	0.45 ± 0.29	0.9 ± 0.82	0.63 ± 0.43	0.56 ± 0.48	0.45 ± 0.27	1.15 ± 0.89
		10 KG	0.45 ± 0.26	0.85 ± 0.88	0.81 ± 0.68	0.4 ± 0.27	0.91 ± 0.79	1.1 ± 0.66
	L5/S	0 KG	0.51 ± 0.3	1.12 ± 0.72	1.24 ± 0.71	0.52 ± 0.36	0.84 ± 1.06	1.6 ± 0.97
		5 KG	0.72 ± 0.54	1.08 ± 0.53	0.43 ± 0.27	0.76 ± 0.52	1.1 ± 0.74	0.54 ± 0.42
		10 KG	1.11 ± 0.88	0.73 ± 1	1.05 ± 0.95	1.08 ± 0.86	1 ± 0.98	0.76 ± 0.53

^**#**^Significant difference between negative recombination at the same level

*Significant difference between left and right translation range at the same level

In the lateral bending movement, the facet joint mainly rotated around the anterior and posterior axes. The average rotation ranges of L3/4, L4/5 and L5/S1 levels under load were 3.2° ± 2.03° to 4.93° ± 4.35°, 1.75° ± 1.24° to 2.6° ± 1.72°, and 0.9° ± 0.85° to 1.75° ± 0.75°, respectively. The data showed that when loading, the rotation range of the L3/4 segment was the largest (*p* < 0.05), and the rotation range gradually decreased from top to bottom (Fig. 5b). The main direction of translation was the craniocaudal direction, which was less than 2.5 mm. We did not find that weight bearing led to significant changes in the kinematics of lumbar facet joints (*p* > 0.05). In addition, for the left and right facets at the same level, the translation range of L3/4 in the craniocaudal direction and anteroposterior direction at 5 kg was greater on the left than on the right (*p* < 0.05). At a 10 kg load, the L5/S1 level was significantly lower on the left than on the right in anteroposterior direction (*p* < 0.05) (Table 1).

In terms of rotational motion, the lumbar facet joint has a more complex motion dominated by coupling motion. The average rotation ranges of L3/4, L4/5 and L5/S1 around the primary rotation axis craniocaudal axis were 1.05° ± 0.8° to 1.86° ± 2.28°, 1.03° ± 0.74° to 1.62° ± 2.32°, and 0.83° ± 0.67° to 1.17° ± 1.11°, respectively. We did not find that weight bearing led to significant changes in the kinematics of lumbar facets (*p* > 0.05), and the data showed that there was no significant difference in the rotation range of the main rotation axis at different horizontal segments (Fig. 5c). In addition, when the left and right facets at the same level were loaded with 5 kg, the left side of the translation range in the front and rear directions of L3/4 was less than that on the right side (left: 0.48 mm ± 0.3, right: 0.81 mm ± 0.45, *p* < 0.05) (Table 1).

## Discussion

In this study, the effects of lifting load (0 kg, 5 kg, 10 kg) on the flexion and extension, left–right bending and left–right rotation of lower lumbar facet joints were measured. In general, the lifting load can affect the coupling displacement of small joints. The data showed that during the flexion and extension exercises, it was found in the mid-lateral direction that the translation range of the L3/4 and L5/S1 segments at 0 kg weight was significantly greater than that at 10 kg (*p* < 0.05).

Overall, the results seem to be the same as those of previous studies. In Chowdhury's [19] study, it was found that the lifting load had a significant impact on LFJ movement, which was reflected in increasing the rotation range and up-down translation range of the main rotating shaft during flexion and extension movement, especially when the lifting load was 13.5 kg. In our study, it was found that the rotation range of the L3/4 and L4/5 levels was also the largest when the load was increased to the maximum 10 kg, but there was no significant difference with other loads. Interestingly, the data in this paper showed that lifting the load could significantly reduce the translation of the external axis. This suggests that physiological load may limit joint activity and discourage excessive activity, which may lead to interfacet impact, thus increasing the risk of facet degeneration. The absence of differences in other kinematics does not mean that weight bearing has no effect on joint kinematics, which may be because weight bearing is not common enough to affect the daily activities of normal adults. However, the literature shows that if the load is too large, such as more than 25 kg, it may become a medium risk factor for low back pain [11].

We found that the horizontal motion patterns of the upper and lower lumbar spines around the main rotation axis are inconsistent in lateral bending. Under 0 kg load and 10 kg load, the rotation range showed a large range at the L3/4 level (*p* < 0.05), and the rotation range gradually decreased from top to bottom. When we tested the subjects' in vivo movements, the pelvis and knee joints of each volunteer were fixed. Therefore, during lateral bending movement, the body center of gravity may be located in the upper segment, and the craniolumbar level is more active. At the same time, we observed that the rotating joint showed strong coupling motion, and the maximum rotation range was not even the main rotation axis but the coupling rotation in the front and rear directions. The possible reason for this result is related to the morphological transformation of facets. The direction of the articular surface is transformed from the sagittal plane and vertical direction to the coronal plane and horizontal direction from top to bottom, which leads to complex coupling motion during rotation. At the same time, this can also confirm that the direction of displacement is mainly in the craniocaudal direction.

From the perspective of LFJ biomechanics, paired facets and intervertebral disks jointly support and stabilize the spine and reduce spinal injury by limiting the movement of all motion planes. This plays a very important role in the load-bearing movement and stability of the whole spine [20]. Under normal human load, LFJs can carry 3–25% of the human load, and the rest is carried by intervertebral disks and ligaments. However, if osteoarthritis occurs, the joint load of abnormal joint force can be as high as 47% [21], which also increases the risk of low back pain. Yin et al. [16] found that with the increase in facet degeneration, the initial increase in lumbar range of motion decreased, which would change the stability of the lumbar spine. Therefore, it is very important to delay facet degeneration. The literature shows that lumbar facet joint asymmetry may be one of the risk factors for intervertebral disk degeneration [22]. In our study, asymmetric displacement of the left and right articular processes was observed in all movements (*p* < 0.05). Any asymmetric displacement of the facet joint will increase the risk of intercartilage impact, leading to osteophyte formation, cartilage erosion, fiber formation or shedding of the facet joint, narrowing of the joint space and nerve foramen, and the formation of synovial cysts, resulting in disorder of the movement mode of the facet joint, resulting in lower back pain.

The findings of this study may have important clinical significance. There is sufficient literature to show that the overweight movement of the lumbar spine will lead to the loss of articular cartilage water, which cannot effectively reduce the impact load and eventually lead to the initial destruction of subchondral bone and osteoarthritis [23]. Therefore, this study can provide accurate data on the internal motion state in the process of joint weight bearing to understand the mechanism of spinal disease and guide us to carry out correct motion postures and reasonable weight-bearing ranges in daily life. At the same time, these data can also provide an important reference for the selection of surgical methods and the design of new spinal implants in lumbar surgery to reduce the risk of adjacent vertebral disease after lumbar surgery. 

## Limitations

Our study also has some limitations. First, the samples in this study were healthy adults. In future studies, relevant pathological control groups should be added to explore the direct relationship between disease and weight bearing. Second, due to the limited visual field, we only studied the range of motion of the L3-S1 horizontal segment. Third, the relationship between the intervertebral disk and facet joint should be discussed. In future, the common changes between them after weight bearing should be further studied by the finite element method. Despite the above limitations, our study still provides accurate data on the in vivo movement of facet joints in different postures under weight-bearing conditions.

## Conclusion

This in vivo study provides us with accurate data on the weight-bearing of facet joints in various body positions. It can guide us how to avoid waist injury due to incorrect weightbearing in our daily life, and provide a theoretical basis for studying the pathological changes of spine diseases, and provide reference for guiding clinical surgical procedures and the selection of new spinal implants.

## Data Availability

Please contact the corresponding author for data requests.

## Abbreviations

LFJs: Lumbar facet joints
DFIS: Dual fluoroscopy imaging system
ROM: Ranges of motion
6DOF: 6 Degrees of freedom
DICOM: Digital Imaging and Communications in Medicine
